# Supplementary material for: Altered Cerebral Curvature in Preterm Infants Is Associated with the Common Genetic Variation Related to Autism Spectrum Disorder and Lipid Metabolism
Source: J Clin Med. 2022 May 31;11(11):3135. doi: 10.3390/jcm11113135 (PMC9181724; doi:10.3390/jcm11113135)
Supplement: Supplementary file 1 [file jcm-11-03135-s001.zip › jcm-1671106-supplementary.pdf]

Journal of
Clinical Medicine

MDPI

Supplementary Materials

Table S1. Genotype effects on brain curvature.

Gene	SNPs	Age-adjusted/FDR									
		Left Hemisphere					Right Hemisphere				
		Lateral Orbitofrontal Gyrus	Medial Orbitofrontal Gyrus	Parahippocampal Gyrus	Posterior Cingulate Gyrus	Superior Temporal Gyrus	Lateral Orbitofrontal Gyrus	Medial Orbitofrontal Gyrus	Parahippocampal Gyrus	Posterior Cingulate Gyrus	Superior Temporal Gyrus
OXTR	rs1042778	0.850/0.982	0.033/0.414	0.899/0.981	0.680/0.765	0.080/0.240	0.098/0.528	<0.001/<0.001	0.394/0.974	0.273/0.734	0.005/0.043
OXTR	rs2268493	0.399/0.857	0.287/0.575	0.749/0.981	0.013/0.034	0.045/0.230	0.100/0.528	0.035/0.079	0.400/0.974	0.260/0.734	0.017/0.057
OXTR	rs53576	0.564/0.890	0.224/0.575	0.547/0.923	0.015/0.034	0.027/0.230	0.175/0.528	0.016/0.075	0.711/0.990	0.260/0.734	0.002/0.041
FADS2	rs174570	0.508/0.857	0.329/0.575	0.819/0.981	0.011/0.034	0.051/0.230	0.193/0.528	0.021/0.075	0.799/0.974	0.386/0.734	0.008/0.043
FADS2	rs174576	0.743/0.912	0.256/0.575	0.911/0.981	<0.001/<0.001	0.023/0.230	0.187/0.528	0.023/0.075	0.736/0.990	0.473/0.749	0.007/0.043
FADS2	rs498793	0.668/0.890	0.751/0.819	0.429/0.827	0.015/0.034	0.041/0.230	0.036/0.528	0.054/0.104	0.933/0.990	0.181/0.734	0.003/0.041
COMT	rs740603	0.488/0.857	0.588/0.756	0.703/0.981	0.012/0.034	0.143/0.322	0.349/0.628	0.002/0.027	0.905/0.990	0.384/0.734	0.010/0.045
COMT	rs165774	0.983/0.983	0.121/0.575	0.895/0.981	<0.001/<0.001	0.063/0.240	0.094/0.528	0.024/0.075	0.760/0.990	0.173/0.734	0.015/0.057
COMT	rs174696	0.429/0.857	0.555/0.749	0.722/0.981	0.047/0.098	0.101/0.273	0.349/0.628	0.024/0.075	0.136/0.974	0.324/0.734	0.037/0.111

SNP, single-nucleotide polymorphism; *OXTR*, oxytocin receptor; *FADS2*, fatty acid desaturase 2; *COMT*, catechol-o-methyltransferase; FDR, false discovery rate.

Table S2. Genotype effects on brain volume.

Gene	SNPs	Age-adjusted/FDR				
		Total Gray Matter Volume	Left Hemisphere Cortical Gray Matter Volume	Right Hemisphere Cortical Gray Matter Volume	Left Hemisphere Cerebral White Matter Volume	Right Hemisphere Cerebral White Matter Volume
OXTR	rs1042778	0.748/0.805	0.721/0.972	0.768/0.839	0.703/0.992	0.950/0.995
OXTR	rs2268493	0.730/0.805	0.972/0.972	0.548/0.748	0.901/0.992	0.816/0.995
OXTR	rs53576	0.805/0.805	0.821/0.972	0.839/0.839	0.992/0.992	0.995/0.995
FADS2	rs174570	0.698/0.805	0.876/0.972	0.582/0.748	0.682/0.992	0.825/0.995
FADS2	rs174576	0.613/0.805	0.750/0.972	0.513/0.748	0.954/0.992	0.675/0.995
FADS2	rs498793	0.196/0.805	0.428/0.972	0.267/0.748	0.321/0.992	0.125/0.995
COMT	rs740603	0.377/0.805	0.369/0.972	0.388/0.748	0.760/0.992	0.809/0.995
COMT	rs165774	0.583/0.805	0.772/0.972	0.492/0.748`	0.413/0.992	0.488/0.995
COMT	rs174696	0.314/0.805	0.265/0.972	0.164/0.748	0.689/0.992	0.452/0.995

SNP, single-nucleotide polymorphism; *OXTR*, oxytocin receptor; *FADS2*, fatty acid desaturase 2; *COMT*, catechol-o-methyltransferase; FDR, false discovery rate.

Table S3. Genotype effects on brain volume in the preterm and control groups controlling for age at scan.

Gene	SNPs	Age-adjusted/P_{FDR}				
		Total Gray Matter Volume	Left Hemisphere Cortical Gray Matter Volume	Right Hemisphere Cortical Gray Matter Volume	Left Hemisphere Cerebral White Matter Volume	Right Hemisphere Cerebral White Matter Volume
OXTR	rs1042778	0.113/0.848	0.494/0.943	0.271/0.933	0.026/0.330	0.019/0.304
OXTR	rs2268493	0.272/0.848	0.835/0.943	0.575/0.933	0.083/0.330	0.079/0.304
OXTR	rs53576	0.151/0.848	0.705/0.943	0.284/0.933	0.072/0.330	0.064/0.304
FADS2	rs174570	0.188/0.848	0.751/0.943	0.349/0.943	0.110/0.330	0.072/0.304
FADS2	rs174576	0.189/0.848	0.632/0.943	0.364/0.943	0.109/0.330	0.068/0.304
FADS2	rs498793	0.201/0.848	0.697/0.943	0.336/0.933	0.083/0.330	0.079/0.304
COMT	rs740603	0.332/0.848	0.908/0.943	0.476/0.933	0.158/0.388	0.140/0.378
COMT	rs165774	0.073/0.848	0.362/0.943	0.135/0.933	0.078/0.330	0.068/0.304
COMT	rs174696	0.323/0.848	0.898/0.943	0.466/0.933	0.089/0.330	0.090/0.304

SNP, single-nucleotide polymorphism; *OXTR*, oxytocin receptor; *FADS2*, fatty acid desaturase 2; *COMT*, catechol-o-methyltransferase; FDR, false discovery rate.

Table S4. Genotype effects on brain thickness.

Gene	SNPs	Age-adjusted/FDR									
		Left Hemisphere					Right Hemisphere				
		Lateral Orbitofrontal	Medial Orbitofrontal	Parahippocampal	Posterior Cingulate	Superior Temporal	Lateral Orbitofrontal	Medial Orbitofrontal	Parahippocampal	Posterior Cingulate	Superior Temporal
OXTR	rs1042778	0.210/0.962	0.031/0.155	0.296/0.920	0.371/0.669	0.113/0.917	0.253/0.885	0.869/0.869	0.497/0.688	0.682/0.682	0.044/0.440
OXTR	rs2268493	0.855/0.962	0.989/0.989	0.368/0.920	0.468/0.669	0.751/0.917	0.826/0.885	0.222/0.740	0.529/0.688	0.299/0.682	0.315/0.525
OXTR	rs53576	0.996/0.996	0.512/0.853	0.332/0.920	0.343/0.669	0.569/0.917	0.885/0.885	0.456/0.869	0.799/0.888	0.302/0.682	0.957/0.957
FADS2	rs174570	0.851/0.962	0.839/0.989	0.789/0.957	0.920/0.920	0.558/0.917	0.749/0.885	0.832/0.869	0.095/0.605	0.575/0.682	0.296/0.525
FADS2	rs174576	0.610/0.962	0.471/0.853	0.697/0.957	0.764/0.849	0.287/0.917	0.787/0.885	0.768/0.869	0.121/0.605	0.540/0.682	0.215/0.525
FADS2	rs498793	0.491/0.962	0.932/0.989	0.663/0.957	0.310/0.669	0.780/0.917	0.411/0.885	0.681/0.869	0.499/0.688	0.469/0.682	0.248/0.525
AP0E	rs405509	0.833/0.962	0.958/0.989	0.171/0.920	0.055/0.550	0.536/0.917	0.441/0.885	0.704/0.869	0.550/0.688	0.063/0.630	0.857/0.952
COMT	rs740603	0.341/0.962	0.380/0.853	0.979/0.979	0.609/0.761	0.903/0.917	0.482/0.885	0.106/0.555	0.266/0.688	0.269/0.682	0.416/0.594
COMT	rs165774	0.682/0.962	0.463/0.853	0.861/0.957	0.444/0.669	0.917/0.917	0.831/0.885	0.427/0.869	0.371/0.688	0.675/0.682	0.787/0.952
COMT	rs174696	0.055/0.962	0.031/0.155	0.723/0.957	0.117/0.585	0.521/0.917	0.041/0.410	0.111/0.555	0.940/0.940	0.420/0.682	0.146/0.525

SNP, single-nucleotide polymorphism; *OXTR*, oxytocin receptor; *FADS2*, fatty acid desaturase 2; *COMT*, catechol-o-methyltransferase; FDR, false discovery rate.

Table S5. Genotype effects on brain curvature in the preterm and control groups controlling for age at scan.

Gene	SNPs	Age-adjusted/FDR									
		Left Hemisphere					Right Hemisphere				
		Lateral Orbitofrontal Gyrus	Medial Orbitofrontal Gyrus	Parahippocampal Gyrus	Posterior Cingulate Gyrus	Superior Temporal Gyrus	Lateral Orbitofrontal Gyrus	Medial Orbitofrontal Gyrus	Parahippocampal Gyrus	Posterior Cingulate Gyrus	Superior Temporal Gyrus
OXTR	rs1042778	0.259/0.957	0.044/0.531	0.276/0.827	0.387/0.992	0.112/0.833	0.317/0.914	0.980/0.981	0.644/0.820	0.788/0.851	0.070/0.545
OXTR	rs2268493	0.990/0.994	0.581/0.977	0.062/0.698	0.350/0.992	0.270/0.833	0.880/0.968	0.911/0.981	0.432/0.807	0.295/0.556	0.456/0.663
OXTR	rs53576	0.427/0.957	0.712/0.977	0.139/0.698	0.948/0.992	0.676/0.879	0.417/0.914	0.723/0.981	0.296/0.807	0.303/0.556	0.150/0.545
FADS2	rs174570	0.695/0.994	0.673/0.977	0.409/0.893	0.961/0.992	0.520/0.877	0.486/0.914	0.941/0.981	0.482/0.807	0.412/0.654	0.198/0.545
FADS2	rs174576	0.494/0.957	0.881/0.977	0.562/0.972	0.992/0.992	0.906/0.986	0.385/0.914	0.871/0.981	0.614/0.820	0.615/0.751	0.232/0.545
FADS2	rs498793	0.994/0.994	0.631/0.977	0.329/0.827	0.694/0.992	0.528/0.877	0.968/0.968	0.959/0.981	0.408/0.807	0.438/0.657	0.241/0.545
COMT	rs740603	0.906/0.994	0.453/0.977	0.302/0.827	0.888/0.992	0.395/0.833	0.491/0.914	0.789/0.981	0.368/0.807	0.223/0.556	0.263/0.545
COMT	rs165774	0.601/0.994	0.882/0.977	0.648/0.972	0.822/0.992	0.986/0.986	0.338/0.914	0.639/0.981	0.546/0.819	0.513/0.693	0.182/0.545
COMT	rs174696	0.897/0.994	0.327/0.977	0.273/0.827	0.724/0.992	0.313/0.833	0.876/0.968	0.877/0.981	0.458/0.807	0.103/0.556	0.220/0.545

SNP, single-nucleotide polymorphism; *OXTR*, oxytocin receptor; *FADS2*, fatty acid desaturase 2; *COMT*, catechol-o-methyltransferase; FDR, false discovery rate.

Table S6. Significance of minor allele frequencies of SNPs and GA.

		Early Preterm (n = 9)	Late Preterm (n = 11)	Control (n = 6)	Pearson χ^2
OXTR	rs1042778	0	1	1	0.409
OXTR	rs2268493	3	3	0	0.571
OXTR	rs53576	5	7	3	0.650
FADS2	rs174570	5	7	4	0.511
FADS2	rs174576	5	7	5	0.569
FADS2	rs498793	5	1	0	0.102
COMT	rs740603	6	8	3	0.729
COMT	rs165774	2	3	1	0.985
COMT	rs174696	6	9	3	0.165

SNP, single-nucleotide polymorphism; *OXTR*, oxytocin receptor; *FADS2*, fatty acid desaturase 2; *COMT*, catechol-o-methyltransferase; GA, gestational age.

Table S7. The sex differences of cerebral development.

Characteristics	Male (n = 15)	Female (n = 11)	p-Value
Volume			
Total gray matter volume	107825.85 ± 13431.40	106143.82 ± 16315.27	0.776
Left hemisphere cortical gray matter volume	36660.81 ± 4637.06	36482.69 ± 6379.82	0.935
Right hemisphere cortical gray matter volume	36231.77 ± 4603.25	34957.95 ± 5693.85	0.534
Left hemisphere cerebral white matter volume	73300.92 ± 10143.10	65353.17 ± 8864.55	0.048
Right hemisphere cerebral white matter volume	69638.25 ± 7685.66	63059.96 ± 8244.22	0.047
Thickness			
Left lateral orbitofrontal gyrus	1.08 ± 0.14	1.18 ± 0.23	0.209
Right lateral orbitofrontal gyrus	1.07 ± 0.16	1.09 ± 0.17	0.708
Left medial orbitofrontal gyrus	1.13 ± 0.12	1.19 ± 0.19	0.371
Right medial orbitofrontal gyrus	1.18 ± 0.11	1.20 ± 0.23	0.749
Left parahippocampal gyrus	1.05 ± 0.16	1.05 ± 0.17	0.979
Right parahippocampal gyrus	1.05 ± 0.14	1.01 ± 0.27	0.671
Left posterior cingulate gyrus	1.33 ± 0.13	1.33 ± 0.15	0.909
Right posterior cingulate gyrus	1.32 ± 0.13	1.37 ± 0.10	0.248
Left superior temporal gyrus	1.40 ± 0.11	1.41 ± 0.19	0.923
Right superior temporal gyrus	1.42 ± 0.11	1.40 ± 0.21	0.814
Curvature			
Left lateral orbitofrontal gyrus	1.95 ± 0.57	2.08 ± 0.68	0.595
Right lateral orbitofrontal gyrus	2.11 ± 0.59	2.10 ± 0.68	0.962
Left medial orbitofrontal gyrus	1.56 ± 0.41	1.69 ± 0.62	0.531
Right medial orbitofrontal gyrus	1.68 ± 0.56	1.49 ± 0.46	0.367
Left parahippocampal gyrus	0.50 ± 0.29	0.62 ± 0.20	0.267
Right parahippocampal gyrus	0.54 ± 0.44	0.35 ± 0.24	0.204
Left posterior cingulate gyrus	0.92 ± 0.44	0.74 ± 0.27	0.220
Right posterior cingulate gyrus	0.90 ± 0.28	0.86 ± 0.41	0.753
Left superior temporal gyrus	5.27 ± 0.99	4.87 ± 0.80	0.284
Right superior temporal gyrus	4.53 ± 0.99	4.38 ± 0.88	0.698
